# Congenital hypothyroidism impairs spine growth of dentate granule cells by downregulation of CaMKIV

**DOI:** 10.1038/s41420-021-00530-z

**Published:** 2021-06-14

**Authors:** Qingying Tang, Shuxia Chen, Hui Wu, Honghua Song, Yongjun Wang, Jinlong Shi, Youjia Wu

**Affiliations:** 1 Department of Pediatrics, Affiliated Hospital of Nantong University, Nantong, Jiangsu Province China; 2 Department of Pediatrics, The Fourth Affiliated Hospital of Nantong University, Yancheng, Jiangsu Province China; 3 Center of Special Inspection, Affiliated Hospital of Nantong University, Nantong, Jiangsu Province China; 4 Key Laboratory of Neuroregeneration of Jiangsu and Ministry of Education, Co-innovation Center of Neuroregeneration, Nantong University, Nantong, Jiangsu Province China; 5 Department of Neurosurgery, Affiliated Hospital of Nantong University, Nantong, Jiangsu Province China

**Keywords:** Thyroid diseases, Paediatric neurological disorders

## Abstract

Congenital hypothyroidism (CH), a common neonatal endocrine disorder, can result in cognitive deficits if delay in diagnose and treatment. Dentate gyrus (DG) is the severely affected subregion of the hippocampus by the CH, where the dentate granule cells (DGCs) reside in. However, how CH impairs the cognitive function via affecting DGCs and the underlying mechanisms are not fully elucidated. In the present study, the CH model of rat pups was successfully established, and the aberrant dendrite growth of the DGCs and the impaired cognitive behaviors were observed in the offspring. Transcriptome analysis of hippocampal tissues following rat CH successfully identified that calcium/calmodulin-dependent protein kinase IV (CaMKIV) was the prominent regulator involved in mediating deficient growth of DGC dendrites. CaMKIV was shown to be dynamically regulated in the DG subregion of the rats following drug-induced CH. Interference of CaMKIV expression in the primary DGCs significantly reduced the spine density of dendrites, while addition of T3 to the primary DGCs isolated from CH pups could facilitate the spine growth of dendrites. Insights into relevant mechanisms revealed that CH-mediated CaMKIV deficiency resulted in the significant decrease of phosphorylated CREB in DGCs, in association with the abnormality of dendrites. Our results have provided a distinct cell type in hippocampus that is affected by CH, which would be beneficial for the treatment of CH-induced cognitive deficiency.

## Introduction

Congenital hypothyroidism (CH) is one of important pediatric health concerns, as it impacts on child growth and neurodevelopment if delay in early treatment [1, 2]. The CH is resulted from defective thyroid gland development, impaired hormone biosynthesis, or several prenatal maternal factors, such as iodine deficiency and maternal IgG antibody adverse effects on fetus [3–5]. Low levels of total thyroxine (T4) and an increase of serum thyroid-stimulating hormone (TSH) in hypothyroidism resulted in deficiency of biologically active triiodothyronine (T3) in fetus [5–8]. Extensive studies have described that reduction of T3 level exerts profound effects on structural and functional changes within brain, depending on developmental stages [9–11]. T3 deficiency is known to reduce proliferation, survival, and differentiation of neuronal and glial progenitors [12, 13]. In the adult brain, however, T3 perturbation causes depressive behavior and deteriorates cognitive function [11, 14, 15]. It is well established that hippocampus is one of primarily affected brain regions of T3, where a reduction of the thyroid hormone will result in the impairment of neurogenesis, as well as cognitive and behavioral deficits [14, 16]. Although T3-mediated signaling through nuclear receptors TH receptor alpha and beta has been well documented in link with the structural abnormality of hippocampus, such as the downstream Emx2 and Klf9 governing neurogenesis [17, 18], the effects of hypothyroidism on the development of hippocampus and the underlying mechanisms are not fully elucidated.

Dentate gyrus (DG), a subregion of the hippocampus is involved in the memory formation. The developmentally generated and adult-generated dentate granule cells (DGCs) are continuously added to the DG and integrated into hippocampal memory networks [19, 20]. DGCs extend axons along the mossy fiber tract to CA3 and receive excitatory synaptic input from perforant path afferents [21–23]. The proper formation and morphogenesis of DGC dendrites is essential to the establishment of neuronal connectivity [24]. Deficits in dendrite size and complexity have been shown to be associated with the impaired cognitive function or other neurological disorders, such as epilepsy [25, 26]. Previous study has demonstrated that iodine deficiency delays the maturation of newborn granule neurons in DG [27], it is reasonably assumed that CH is able to affect the growth of DGC dendrites, in addition to the proliferation and survival of progenitors.

Calcium/calmodulin-dependent protein kinase IV (CaMKIV) has been shown to regulate the dendrite growth of both cortical and hippocampal neurons via CREB and CREB-binding protein (CBP)-regulated transcription [28–31]. An early study has demonstrated that the promoter of CaMKIV contains T3 response element, and CaMKIV can be regulated directly by T3 in primary cultured neurons from fetal cortex [32, 33]. These imply that CH may affect the dendrite growth of DGCs through regulation of CaMKIV, and eventually contributes to impairment of cognitive function. In the present study, we successfully established CH model of rat fetus and investigated the CH effects on the cognitive function of the animals. We further observed the numbers and dendrite growth of DGCs in CH pups. Subsequent transcriptome profile analysis revealed that CaMKIV acted as a central hub responsible for CH-mediated impairment of DGC dendrite growth during development of hippocampus. Our results have provided new mechanism of CH-mediated deficiency of hippocampal memory circuits.

## Results

### Continuous treatment of MMI on dams results in CH-mediated cognitive deficits of offspring

**Fig. 1:**
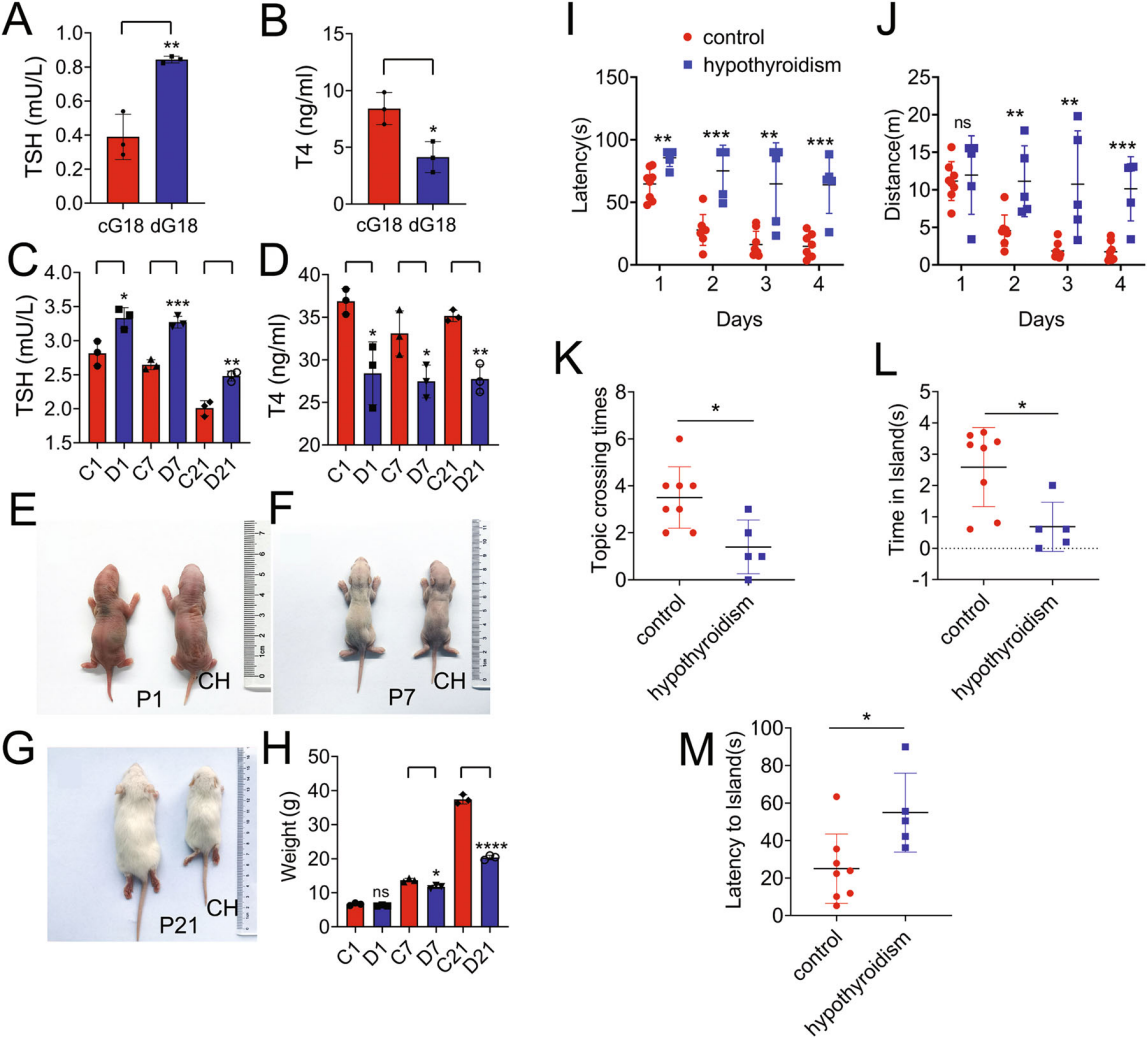
MMI treatment resulted in CH and cognitive deficits of rat offspring. **A**, **B** Measurement of TSH (**A**) and T4 (**B**) in serum of dams at day 18 of gestation following given 0.02% MMI in the drinking water from the day 9 of gestation. *n* = 3. **C**, **D** Measurement of TSH (**C**) and T4 (**D**) in serum of P1, P7, and P21 pups. *n* = 12. Experiments were performed in triplicates. **E**–**H** Body weight determination of P1, P7, and P21 pups following CH. C1/7/21 indicates control, and D1/7/21 indicates CH. *n* = 12. Experiments were performed in triplicates. **I** Morris water maze tests of rat offspring at 24 weeks following CH showed escape latency in the acquisition phase. **J** Swimming distance in the target quadrant in the acquisition phase. **K** Number of target crossings in the probing trial. **L** Times staying in platform in the probing trial. **M** Escape latency in the probing trial. *n* = 5. Error bars represent the standard deviation (*P < 0.05).

Whether maternal thyroid hormones are necessary for normal embryonic development is uncertain until T4 and T3 are detected in the embryonic tissues of rat and human before onset of fetal thyroid function [34]. 2-Mercapto-1-methylimidazole (MMI) has been extensively applied to induce maternal and fetal hypothyroidism [35, 36]. So, it was given in drinking water of rat dams from day 9 of gestation till postnatal day 21 (P21). To assess MMI-induced CH efficiency in fetal rats, the TSH and T4 levels in serum of both dams and pups were thereafter determined. Results showed that TSH contents in dams at day 18 of gestation and the P1, P7, and P21 pups significantly increased following MMI treatment, while T4 levels markedly reduced comparing to the normal groups (Fig. 1A–D). These changes of TSH and T4 levels in serum were in consistent with the clinicopathologic indicators of hypothyroidism. Further phenotype observation displayed that the body weights of CH pups were much lower than those of controls from P7 onward (Fig. 1E–H). Morris water maze test was then performed to evaluate the effects of maternal CH on learning and cognitive function of the offspring at 24 weeks. The escape latencies and swimming distances from CH group were significantly longer than those of control from day 2 to day 4 (Fig. 1I, J). Probe trial test on fifth day for recording the number of times each rat crossing the platform area and the duration time in island, showed that the CH offspring suffered from severe behavioral deficits. Also, the escape latencies at day 5 were significantly longer than those of the control (Fig. 1K–M). The data indicate that CH severely impairs the cognitive function of the rats.

### CH leads to aberrant growth of DGC dendrites in pup hippocampus

**Fig. 2:**
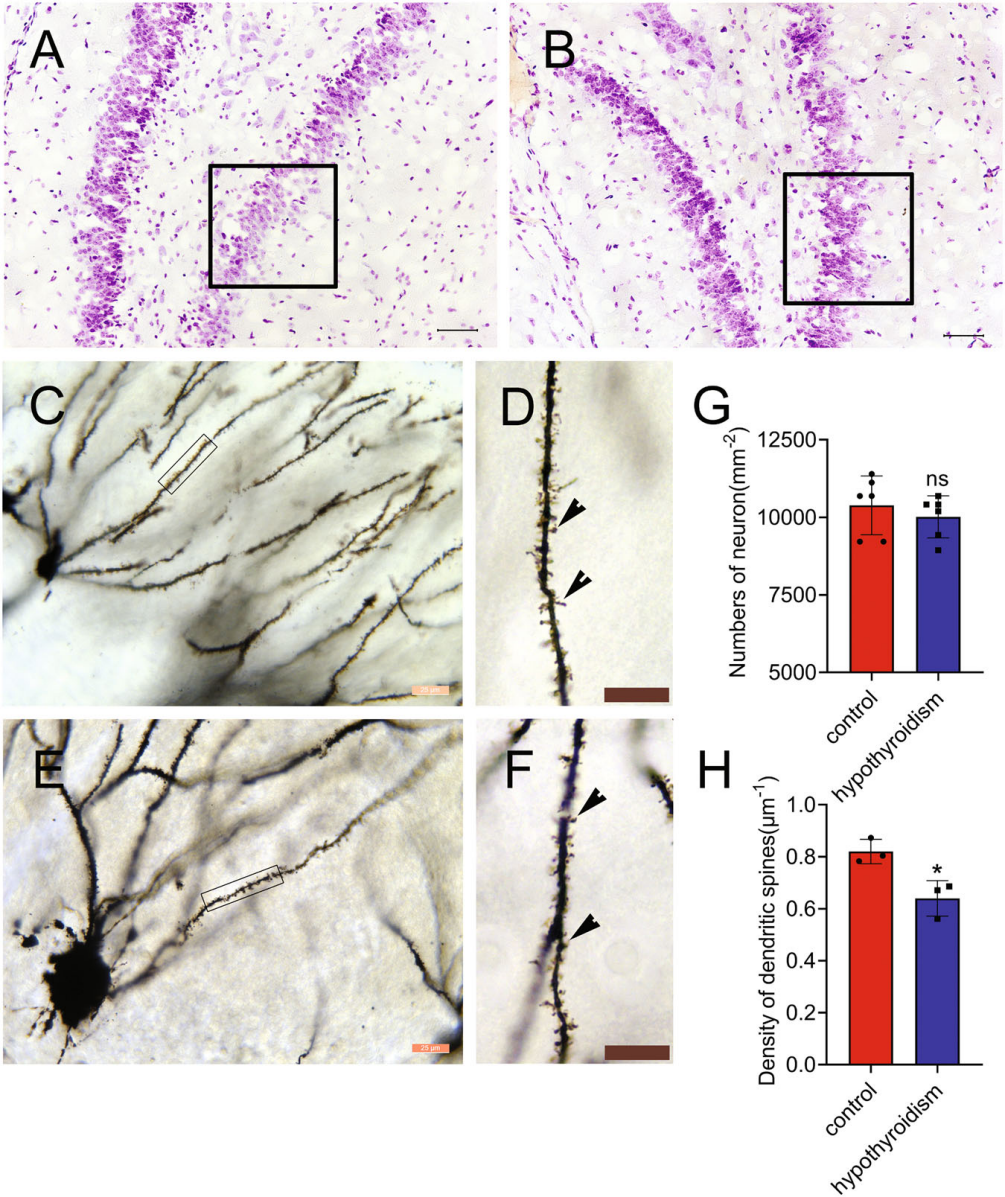
Effects of CH on the spine density of DGCs in the DG subregion of rat pups. **A**, **B** Nissl’s staining of P21 pup hippocampus with or without CH. **C**–**H** Comparison of dendritic spine density of DGCs by Golgi staining between P21 pups with or without CH. The rectangles in **A** and **B** indicate detection sites of dendritic spines in DG subregion. The rectangles in **C** and **E** indicate region magnified in **D** and **F**. **H**, **G** Statistical analysis of **C**, **E** and **A**, **B**, respectively. Arrowheads indicate spines. Statistic analysis of spine density in triplicates each 15 fields. Scale bars, 50 μm in **A**, **B**; 25 μm in **C**, **E**; 10 μm in **D**, **F**. Error bars represent the standard deviation (*P < 0.05).

As integration of new neurons into DG and their maturation are essential for supporting memory networks in hippocampus [20, 37], the number of neurons in DG subregion and the morphology of DGC dendrites in CH pups at different stages were thus observed. Results showed that CH did not affect the number of neurons in the DG of pups, as defined by Nissl staining (Fig. 2A, B, G). However, MMI treatment significantly decreased the density of dendritic spines observed by Golgi staining (Fig. 2C–F, H). The results indicate that CH-mediated cognitive deficits of rat might be involved in the aberrant spine growth of DGC dendrites in pups.

### Molecular aspects of CH action on developing hippocampus of rat

**Fig. 3:**
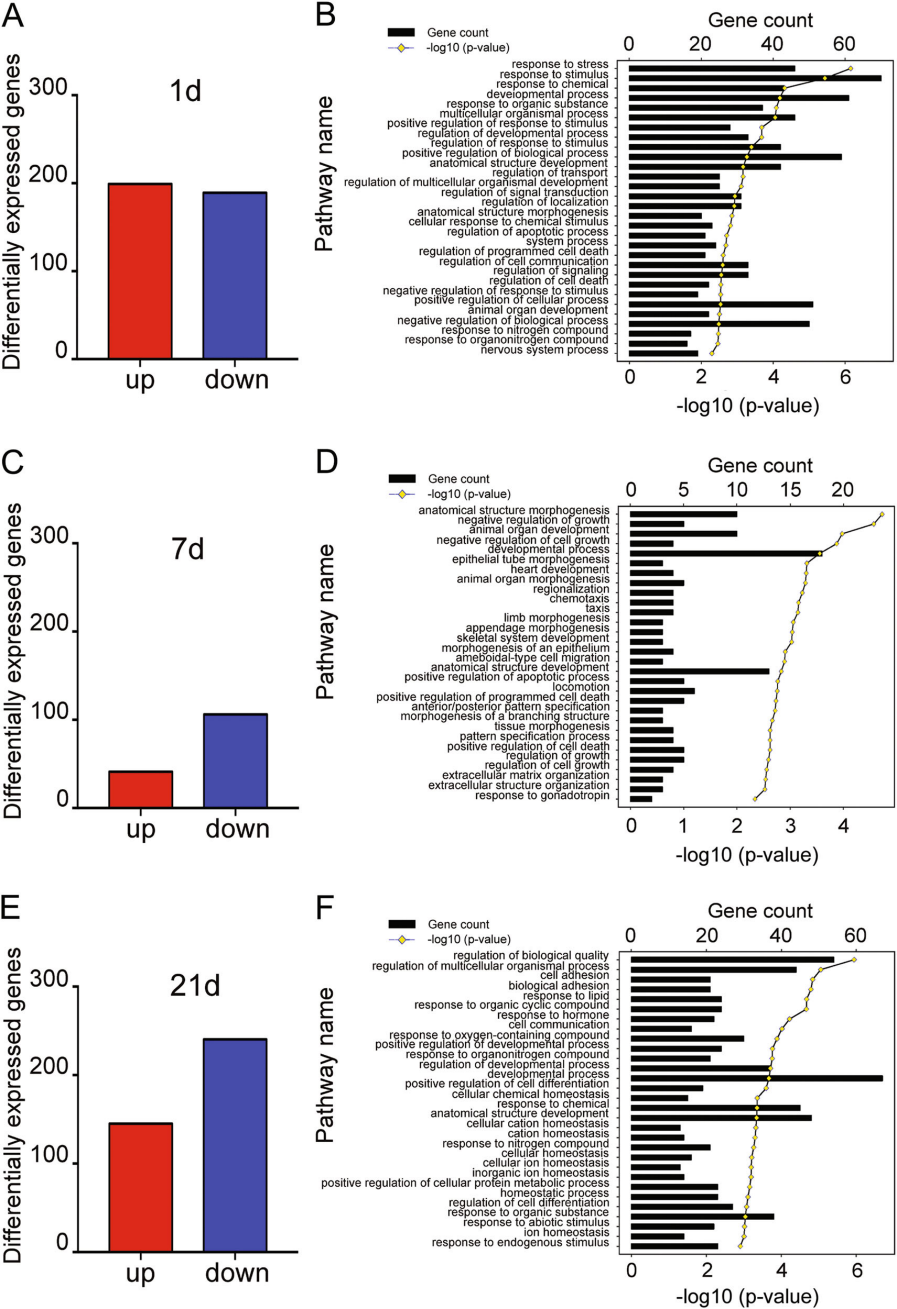
Functional annotations of DEGs in the pup hippocampus following CH. **A**, **C**, **E** Bar graphs of DEGs in the P1, P7, and P21 rat pup hippocampus following CH. **B**, **D**, **F** Most significantly enriched groups for the DEGs relating to biological process by GO annotations. The significance of enrichment is expressed as a −log10 (*P* value).

**Fig. 4:**
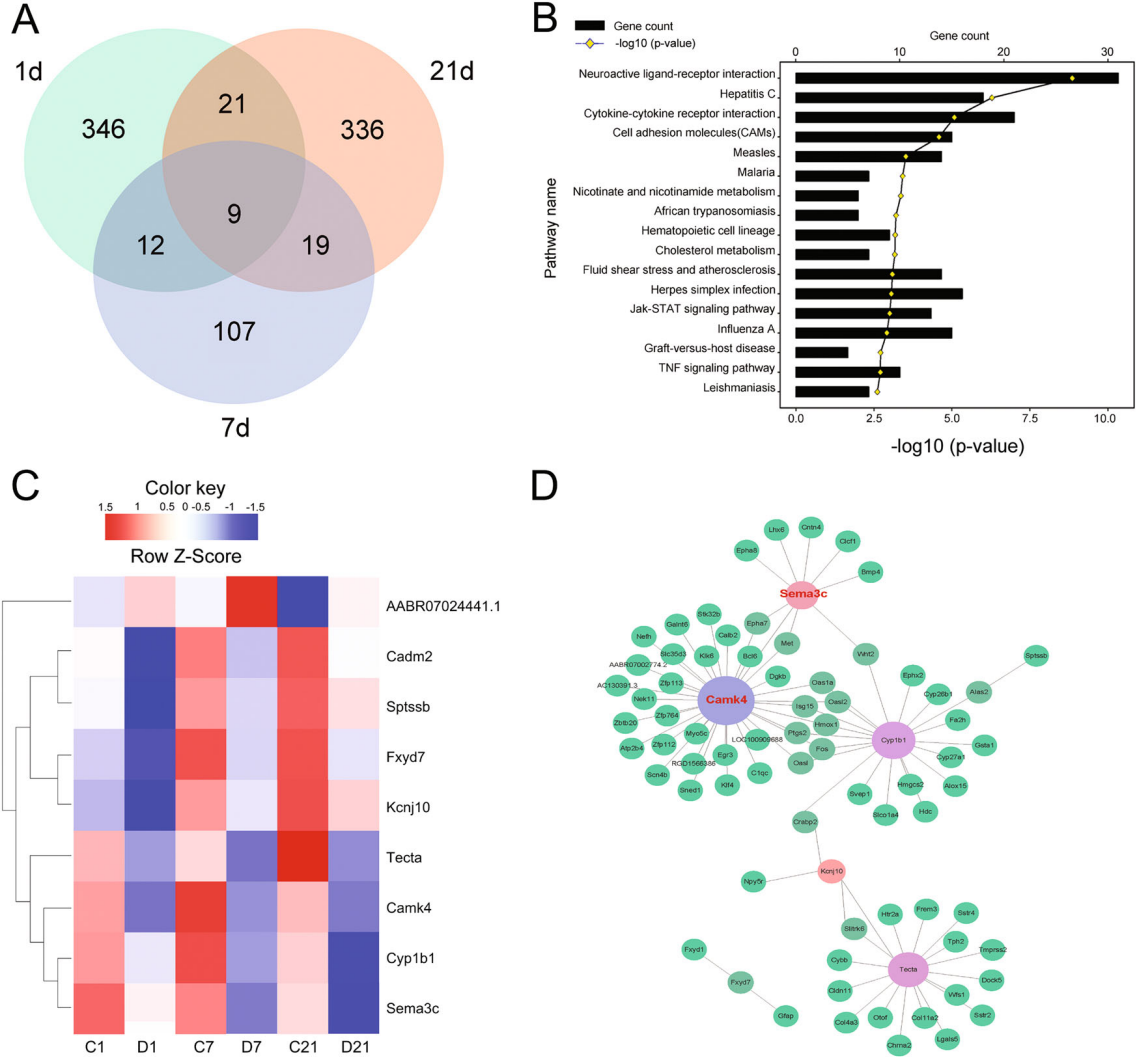
Analysis of key player in mediating aberrant spine growth of DGC in the CH offspring of rat. **A** Integration of DEGs in the hippocampus of P1, P7, and P21 rat pups following CH. **B** Most significantly enriched groups for the integrated DEGs relating to pathways. **C** Heatmap and cluster dendrogram of integrated DEGs in the hippocampus of P1, P7, and P21 rat pups following CH. **D** A reconstructed gene network was created using the ingenuity pathway analysis software (IPA) on the basis of integrated DEGs.

Thyroid hormone, primarily T3, has important effects on the development of rat hippocampus by influence on gene expression in a manner of genomic or nongenomic actions [18, 38]. To reveal the molecular aspects of CH action on the developing hippocampus of rat, which might provide clues involved in abnormality of DGC dendrites, we performed transcriptome analysis of hippocampal tissues dissected from CH pups at P1, P7, and P21, respectively. A total of 388, 147, and 385 differentially expressed genes (DEGs) were identified at the three time points, with defined criteria of *P* < 0.05 and a greater or less than twofold changes (Fig. 3A, C, E and Table S1). The Gene Ontology (GO) enrichment analysis classified these DEGs involved in the biological process of anatomical structure morphogenesis, negative regulation of growth, developmental process, and response to hormone (Fig. 3B, D, F). We further integrated the DEGs at three time points, and characterized nine functional genes associated with important biological processes, including neuroactive ligand–receptor interaction following CH (Fig. 4A, B). These genes displayed dynamic alteration in the hippocampus comparing to the control, as shown by heatmap and cluster dendrogram (Fig. 4C). To elucidate the mechanism of CH impairment on dendritic growth of hippocampal neurons, we performed ingenuity pathway analysis (IPA) for the DEGs integrated at P1, P7, and P21. A gene network was constructed, and as a result identifying that CaMKIV was highlighted as the prominent regulator in response to reduction of thyroid hormone (Fig. 4D). The data indicate that CaMKIV is a potential player in mediating aberrant spine growth of DGC dendrites in the CH offspring.

### CaMKIV is downregulated in the granule cell layer of dentate gyrus following CH of pups

**Fig. 5:**
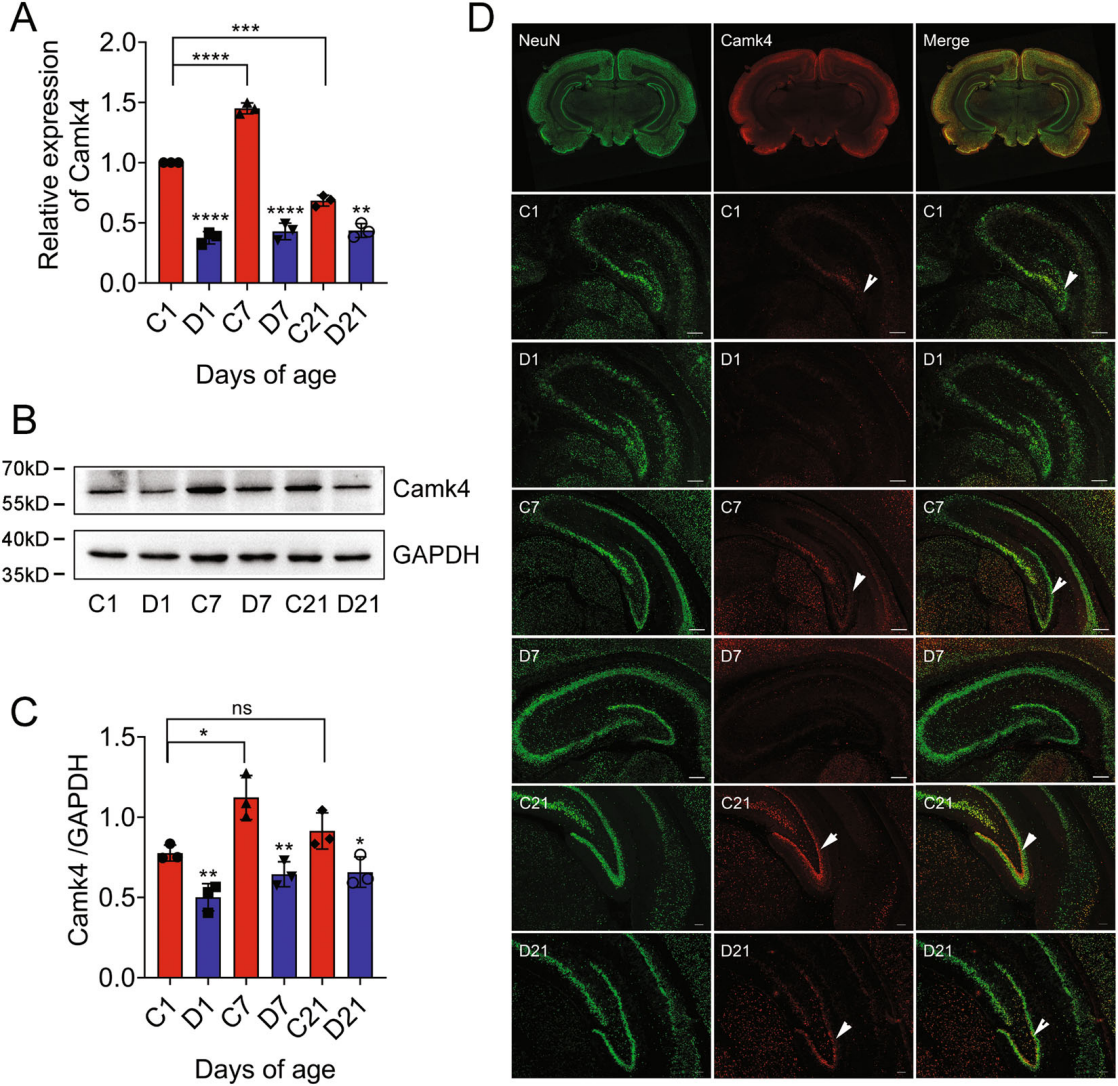
Dynamic expression of CaMKIV in the hippocampus of rat pups following CH. **A** RT-PCR analysis of CaMKIV transcription in the hippocampus of P1, P7, and P21 pups following CH. **B** Western blot analysis of CaMKIV. **C** Statistical analysis of **B**. Experiments were performed in triplicates. Error bars represent the standard deviation (*P* < 0.05). **D** CaMKIV immunostaining of the cross section from hippocampus of P1, P7, and P21 pups following CH. C1/7/21 indicates control, and D1/7/21 indicates CH. Arrowheads indicate positive signals in the dentate gyrus. Scale bars, 200 μm.

To substantiate the inference drawn from transcriptome analysis, both RT-PCR and western blot were performed to examine the expression changes of CaMKIV at transcriptional and translational levels in the hippocampus, following CH of pups. Consistently, expression of CaMKIV in CH neonatal rat was significantly downregulated at P1, P7, and P21, comparing with those in normal group (Fig. 5A–C). Immunofluorescence demonstrated that CaMKIV protein distributed in the DG, CA1, and CA3 regions of hippocampus, with the positive signals markedly reduced in the granule cell layer of DG following CH (Fig. 5D). The data indicate that CH negatively regulates the expression of CaMKIV in the hippocampal DGCs of pups.

### Knockdown of CaMKIV in DGCs is associated with decrease of spine density

**Fig. 6:**
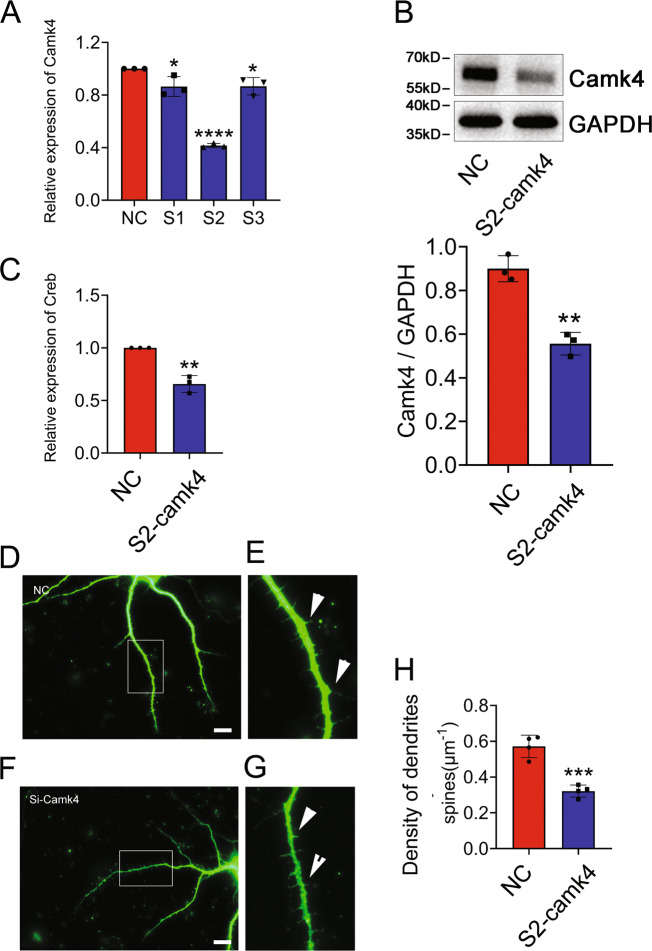
Effects of CaMKIV deficiency on the spine density of DGC dendrites. **A**, **B** Interference efficiency of three siRNA oligonucleotides for CaMKIV was measured by RT-PCR (**A**) and western blot (**B**). The siRNA2 was used for the knockdown experiments. **C** Transcriptional analysis of CREB following CaMKIV knockdown of DGCs for 24 h. **D**–**G** Detection of the spine density of dendrites stained by MAP2 antibody following CaMKIV knockdown of primary DGCs and kept for culture of another 5 days. Scramble was used as control. The rectangle indicates region magnified. **H** Statistic analysis of spine density in quadruplicates each 15 fields. Six pups of each group were used to isolate DGCs. Arrowheads indicate spines. Scale bars, 10 μm.

To elucidate the potential physiological function of CaMKIV reduction in the DGCs, we cultured the primary DGCs, followed by interference of the CaMKIV expression using the siRNA oligonucleotides. The siRNA2 with nearly 60% knockdown efficiency was selected for the subsequent experiments (Fig. 6A, B). Transfection of CaMKIV siRNA2 to the DGCs for 24 h resulted in the decrease of spine density in comparison with the control (Fig. 6D–H). Also, it led to the deficient expression of *creb* (Fig. 6C). These results indicate that CH-mediated downregulation of CaMKIV in the DGCs are implicated in the deficient spine growth of dendrites.

### T3 is able to potentiate spine growth of DGCs from CH pups through regulation of CaMKIV/CREB signaling

**Fig. 7:**
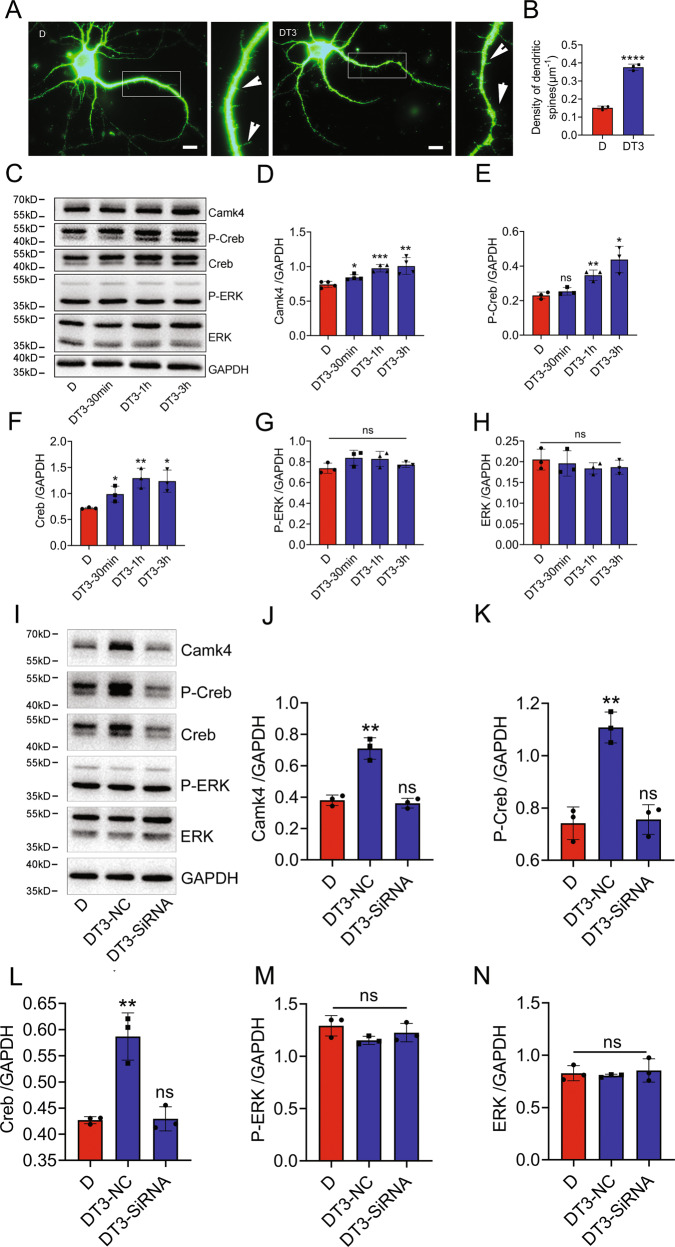
T3 increased spine density of DGCs from CH fetal rat via activation of CREB. **A** DGCs from CH E18 fetus were treated with 5 nM T3 for 24 h, followed by culture for another 7 days. Spines were immunostained by MAP2 antibody. The rectangle indicates region magnified. Arrowheads indicate spines. **B** Statistical analysis of **A**. Spine density was analyzed in quadruplicates each 15 fields. Six pups of each group were used to isolate DGCs. **C**–**H** Western blot analysis of CaMKIV, p-CREB/CREB, and pERK/ERK following DGCs treatment with 5 nM T3 for 30 min, 1 h, and 3 h, respectively. **D**–**H** are statistical analysis of **C**. **I** Western blot analysis of CaMKIV, p-CREB/CREB, and pERK/ERK following DGCs knockdown of CaMKIV for 24 h, and then stimulated with 5 nM T3 for 30 min. **J**–**N** Statistical analysis of **I**. Experiments were performed in triplicates. Error bars represent the standard deviation (*P* < 0.05). Scale bars, 10 μm in **A**.

Given that CH was able to induce spine deficiency of DGCs in vivo, we thus attempted to reverse these harmful effects of CH on the DGCs by the treatment with thyroid hormone T3 in vitro. Addition of 5 nM T3 to the culture medium of primary DGCs from CH P3 pups for 24 h was able to significantly increase the density of spines (Fig. 7A, B). CREB can be directly phosphorylated by CaMKIV and ERK to activate multiple genes essential for neurogenesis, neuronal survival, and plasticity in hippocampus [39]. To examine whether the CaMKIV/CREB signal pathway was involved in the T3-mediated spine growth, the protein level of phosphorylated CREB was thus determined. Results showed that T3 could markedly activate both CaMKIV and CREB kinases, but made no effects on the activation of ERK (Fig. 7C–H). Knockdown of CaMKIV accordingly resulted in the decease of phosphorylated CREB, rather than ERK (Fig. 7I–N). The data indicate that endogenous T3 is able to rescue the spine density of DGC dendrites in CH pups through regulating CaMKIV/CREB signaling.

### CH leads to the aberrant activation of CREB in the hippocampus of pups

**Fig. 8:**
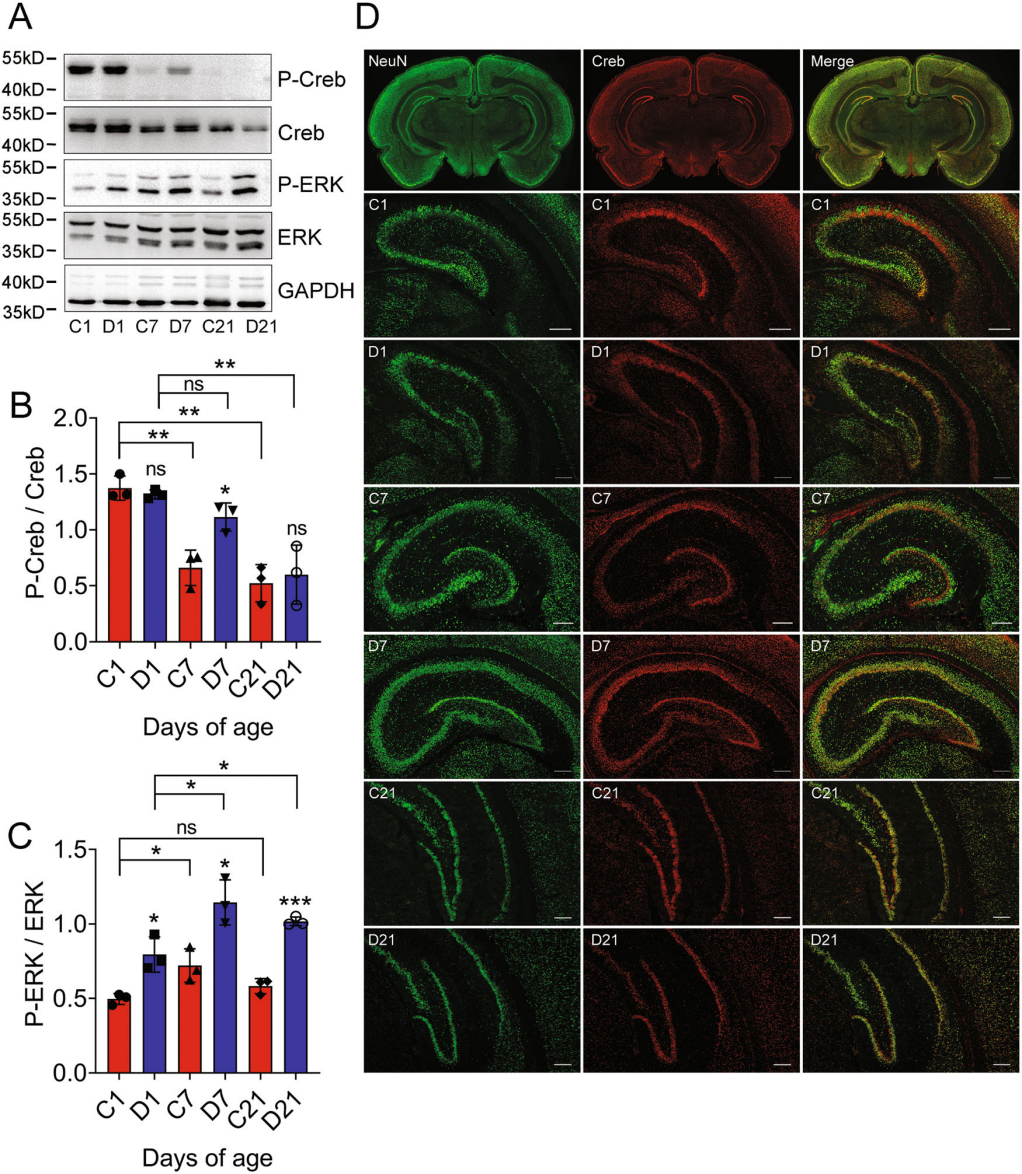
Expression analysis of CREB in the hippocampus of rat pups following CH. **A** Western blot analysis of p-CREB/CREB, pERK/ERK in the hippocampus of P1, P7, and P21 pups following CH. **B**, **C** Quantification data as shown in **A**. Experiments were performed in triplicates. Error bars represent the standard deviation (*P* < 0.05). **D** Immunostaining of CREB protein in the tissue section from hippocampus of P1, P7, and P21 rat pups following CH. Scale bars, 200 μm.

CaMKIV has been found to mediate an early transient phase of CREB phosphorylation, while ERK/MAPK signaling cascade is responsible for a persistent phase of phosphorylation [40]. To understand the regulatory relationship between CaMKIV and CREB proteins in the hippocampus of CH pups, the phosphorylation of CREB in the hippocampal tissues of pups was thus examined at P1, P7, and P21, following dams treatment with MMI. The protein levels of phosphorylated CREB were unexpectedly inconsistent with those of CaMKIV, but in parallel with those of ERK signaling (Fig. 8A–C). Immunostaining demonstrated that CREB was distributed in the multiple neuronal types of hippocampal subregions, in addition to localization in the DGCs (Fig. 8D). The results indicate that CH-induced deficiency of CaMKIV primarily affects spine aberrancy of DGCs by inactivation of CREB in a transient and region-specific manner.

## Discussion

CH is the most common neonatal endocrine disorder, with the incidence between 1:2000 and 1:4000 births [41]. CH is caused by thyroid dysgenesis (primary CH) or impaired TSH-mediated stimulation of the thyroid gland (central CH) [1, 42]. However, maternal hypothyroxinemia can also bring in fetal thyroid hormone deficiency during the embryo development [43–45]. A plethora of evidence suggests that thyroid hormone is essential for normal development, growth, and metabolic regulation of the organs, especially for the neurogenesis and differentiation in CNS [46, 47]. Screening of CH is routinely based on elevated levels of TSH and/or decreased concentrations T4 in serum of infants [8]. In the present study, we established CH model of rat pups by constantly supplying MMI in the drinking water of dams from day 9 of gestation. Measurement of TSH and T4 concentration in the serum of pups confirmed a successful preparation of the CH offspring, by which the impaired cognitive function was objectively evaluated. Several earlier studies have mentioned that thyroid hormones are not required for normal embryonic development before onset of fetal thyroid function. We are herein in favor of the views that maternal hypothyroxinemia is correlated with developmental disorders of neurological system [48].

DGCs are bipolar neurons with dendrite extending into the molecular layer and axon entering the hilar region of hippocampus. The dendrites receive afferent input from fibers of the lateral and medial entorhinal cortex in the outer molecular layer, and from commissural/associational fibers in the inner molecular layer [49, 50]. So, the normal morphology of the DGCs dendrites is developmentally essential for the cognitive function of the offspring, because they connect with both cortical and hippocampal internal input [51]. In fact, the aberrant DGC dendrites have been found to impair long-term spatial memory, temporal order memory, and result in seizure activity by forming an abnormal excitatory feedback circuit [52, 53]. Although granule cells are relatively resistant to a variety of neurological insults [54], several evidences have shown that they are selectively vulnerable to the reduction of circulating hormones, such as adrenal hormones [55, 56]. As such, it is not surprising that CH of pups results in abnormal development of DGC dendrites, which in turn affects cognitive function of the offspring.

Members of the CaMK family are classified into two classes based on their function, the multifunctional CaMKs that each has several substrates (CaMKK, CaMKI, CaMKII, and CaMKIV) and the substrate-specific CaMKIII [57]. Among these members, CaMKII and CaMKIV have extensively been shown to play critical roles in the regulation of neuronal plasticity and cognitive functions [30, 58]. As for CaMKII, its expression level exceeds 1% of total protein in hippocampus and neocortex [58], but it seems less sensitive to the absence of thyroid hormones in comparison with CaMKIV [59]. The protein of CaMKIV is observed to be highly expressed in the cerebral cortex, cerebellum, and hippocampus. Its deficiency exerts a severe influence on the brain development [30, 60, 61]. The primary action of CaMKIV on cortical neurons attributes to mediating dendrite complexity via the regulation of specific morphological features of the dendrite arbor [29, 30]. In the present study, we demonstrated that CH-induced deficiency of CaMKIV significantly reduced the spine density of DGCs, suggesting the conservative function of CaMKIV in regulating dendrite growth of multiple types of neurons.

As a prototypical stimulus-inducible transcription factor, CREB within neurons is responsive to different physiological stimuli. Activation of CREB immediately initiates gene transcription, contributing to proliferation of neuronal precursors, as well as the survival, synaptic connectivity of developing neurons, and the learning and memory of the adult animals [62–65]. The Ser133 in KID domain of CREB is phosphorylated by activators, making CBP bind to facilitate neuronal activity-related gene expression, such as c-fos, egr1, and egr3 [66, 67]. CREB has been detected in multiple regions of the brain at various developmental stages [65]. A number of kinases, including CaMKIV, PKA, AKT, and ERK, are associated with the phosphorylation of CREB with distinct phases of activation [68, 69]. For example, CaMKIV mediates a transient, whereas ERK mediates a sustained phosphorylation of CREB [70]. In the present study, interference of CaMKIV in the DGCs could result in changes of CREB phosphorylation at 30 min, suggesting a transient regulatory function of the kinase. Interestingly, CH-mediated CaMKIV deficiency failed to reduce protein levels of phosphorylated CREB at hippocampus of pups at P1, P7, and P21. The reason is probably attributed to the action of multiple factors on the CREB, as several hormones, neurotransmitters, growth factors, and neurotrophins are able to facilitate CREB phosphorylation during the development of hippocampus [65]. However, the conclusion needs to be further clarified.

In conclusion, CH decreased the expression of CaMKIV kinase in the DGCs of the rat pups, which resulted in the reduced spine density of DGCs dendrites, and as a result, the cognitive impairment of the offspring.

## Materials and methods

### Animals

Adult female Sprague–Dawley (SD) rats, weighing 180–220 g, were provided by the Center of Experimental Animals, Nantong University. All animal experiments were approved by the Animal Care and Use Committee of Nantong University and the Animal Care Ethics Committee of Jiangsu Province. All rats were housed in standard cages (five rats in each cage) in an environment maintained at 22 ± 2 °C on a 12–12 h light–dark cycle, and had free access to water and food.

### Establishment of CH model of rat pups

To induce CH of rat pups, the pregnant dams were given 0.02% MMI in the drinking water from embryonic day 9 (E9, the day of appearance of the vaginal plug was E0) until P21 of the pups. The dams of the control group were given clean drinking water. The concentration of TSH and T4 in the serum of P1, P7, and P21 pups and dams at day 18 of gestation was measured to verify a successful preparation of the CH model.

### Determination of TSH and T4 concentrations

All blood samples, including dams and pups at P1, P7, and P21, were collected and centrifuged at 3000 r.p.m. for 20 min. The upper serum was extracted and processed for TSH and T4 determination, according to the protocol provided by the manufacturer (Enzyme-linked Biotechnology, China). Briefly, 50 μl of standard or sample was added to the appropriate wells, followed by addition of 100 μl of enzyme conjugate. After incubation at 37 °C for 60 min, the incubation mixture was removed and fill each well completely with wash solution (1×). The procedure was repeated for four times, and the plate was inverted for natural dry until no appearance of moisture. Then, 50 μl of substrate A and 50 μl of substrate B were added to each well. After gently mixture, they were allowed to incubate at 37 °C for 15 min avoiding of light. Finally, 50 μl of stop solution was added, and the optical density was read at 450 nm using a microtiter plate reader within 15 min.

### Morris water maze tests

The Morris water maze test was used to measure the learning and memory abilities of the offspring. After establishment of the CH model at 24 weeks, the rats were trained for 4 days each four times. In the place navigation test, the rats were put into the water facing the pool wall (back to platform) with an entry point, to observe and record the time, and distance needed to find and climb onto the platform (escape latency and distance). If the rats did not find the platform within 90 s, they were guided to the platform, and the escape latency was recorded as 90 s. The escape latencies of rats were recorded on days 1–4. In the spatial probe test, the platform was removed on day 5, and the times of crossing the original platform location in the pool, duration, and latency within 90 s were recorded. Data collection and processing were performed using a Morris water maze image automatic monitoring system.

### Golgi staining

Golgi staining was performed according to the instruction of Hito Golgi staining kit (HTKNS1125NH). Briefly, the dissected hippocampus from P21 pup was impregnated at room temperature for 2 weeks in a mixture of solution A and solution B, then immersed in solution C at 4 °C for 48 h. The hippocampus was sectioned into 100-μm slices and transferred onto 2% gelatin-coated slides. The slices were dried at room temperature for 2 days and then stained with a mixture of solution D, solution E, and distilled water (1:1:3 ratio) for 10 min, followed by dehydration with gradient ethanol. The samples were subsequently cleared by xylene and mounted with Permount Medium (Fisher Scientific, Pittsburgh, PA). Sections were observed under the microscope, and the morphology of the dendrites was photographed.

### Nissl’s staining

The freshly dissected hippocampus of P21 pup was sectioned at 12 μm. The slices were dried at room temperature for 30 min, followed by immersing with distilled water. The slices were stained in cresyl violet solution for 30 min, and then were dehydrated with gradient alcohol. After clearing with xylene, they were mounted with undiluted xylene based resinous mounting medium and observed under microscope.

### Primary culture of dentate granule cells and siRNA transfection

Cultures of dissociated granule cells were prepared from P3 to P4 SD rats, as previously described [71]. Briefly, the DG were dissociated in ice-cold Gey’s balanced salt solution in the presence of d-glucose (6.50 g/l), followed by digestion with 0.125% trypsin and 0.01% DNase I at 37 °C for 20 min. The horse serum was then added to stop the digestion, and the cells were suffered to centrifuge at 1000 r.p.m. for 4 min. After removing the supernatants, the cells were dispersed in 2 ml of culture medium with arabinofuranosyl cytidine and serum at 37 °C. For primary cultures, the dissociated granule cells were plated onto 13-mm cover slips coated with poly-d-lysine at a cell density of 1 × 10^4^ cells/cm^2^ in culture medium in 24-well plates, and incubated at 37 °C in a humidified 5% CO_2_ and 95% air atmosphere.

For siRNA transfection of the DGCs, it is achieved by the protocol of the manufacturer (RiboBio, China). Briefly, 120 μl of 1× riboFECT™ CP buffer was added to 15 μl of 20 μM siRNA storage solution, followed by the addition of 12 μl of riboFECT™ CP reagent. After gently mixture and subsequent incubation at room temperature for 15 min, the transfection complex was added into 2 × 10^6^ DGCs cultured in neurobasal medium (final volume 2 ml) in the six-well plates with final concentration of siRNA at 150 nM, and incubated at 37 °C in a humidified 5% CO_2_ and 95% air atmosphere for 24 h. The transfection efficiency of siRNA was accordingly measured.

### Western blot

Proteins were extracted from neuronal cultures or hippocampus with a buffer containing 1% SDS, 100 mmol/l Tris–HCl, and 1 mmol/l PMSF. The protein concentration of each specimen was assessed by the BCA method to maintain the same loads. The protein extracts were heat-denatured at 95 °C for 5 min, electrophoretically separated on 10% SDS–PAGE, and transferred to PVDF membranes. The membranes were reacted with a 1:1000 dilution of primary antibodies in TBST buffer at 4 °C overnight, followed by reaction with a secondary antibody conjugated with goat-anti-rabbit or donkey-anti-mouse HRP (Proteintech), diluted at 1:1000 at room temperature for 2 h. After the membrane was washed, the HRP activity was detected using an ECL kit. Antibodies used for western blot were anti-CaMKIV antibody (1:1000; proteintech); anti-CREB antibody (1:1000; proteintech); anti-p-CREB antibody (1:1000; CST); anti-ERK antibody (1:1000; CST); anti-p-ERK antibody (1:1000; CST); and anti-GAPDH antibody (1:1000; CST).

### Transcriptome sequencing of pup hippocampus and bioinformatics analysis

Total RNA of hippocampus from P1, P7, and P21 pups with or without CH, was extracted using the mirVana miRNA Isolation Kit (Ambion, Austin, TX), according to the manufacturer’s instructions. They were then selected by RNA Purification Beads (Illumina, San Diego, CA), and undergone library construction and RNA-seq analysis. The library was constructed by using the Illumina TruSeq RNA sample Prep Kit v2 and sequenced by the Illumina HiSeq-2000 for 50 cycles. High-quality reads that passed the Illumina quality filters were kept for the sequence analysis.

Differentially expressed mRNA was designated in a criterion of greater or less than twofold change in comparison with control. Function of genes was annotated by Blastx against the NCBI database or the AGRIS database (https://agris-knowledgebase.org/) with *E* value threshold of 10^−5^. GO classification was obtained by WEGO (http://wego.genomics.org.cn/) via GO id annotated by Perl and Rprogram. Kyoto Encyclopedia of Genes and Genomes (KEGG) pathways were assigned to the sequences using KEGG Automatic Annotation Server online. For all heatmaps, genes were clustered by Jensen–Shannon divergence. A reconstructed gene network was created using the IPA software on the basis of DEGs to investigate their regulatory pathways and cellular functions [72].

### Quantitative real-time polymerase chain reaction (Q-PCR)

Total RNA was prepared with TRIzol (Gibco) from hippocampus or neuronal cultures upon demands. The first-strand cDNA was synthesized using the Omniscript reverse transcription kit (Qiagen) in a 20-µl reaction system containing 2 µg total RNA, 0.2 U/µl M-MLV reverse transcriptase, 0.5 mmol/l dNTP mix, and 1 µmol/l Oligo-dT primer. The cDNA was diluted 1:4 before use in Q-PCR assays. The sequence-specific primers were designed and synthesized by Generay (Shanghai, China): for CaMKIV, forward primer 5′-TGG AGG CAG TTG CTT ACC TG-3′ and reverse primer 5′-CCT CGG AGA ATC TCA GGT GC-3′; for CREB, forward primer 5′-GCA GTG ACT GAG GAG CTT GT-3′ and reverse primer 5′-ACC TGG GCT AAT GTG GCA AT-3′. The reactions were performed using one initial denaturation cycle at 94 °C for 5 min followed by 45 cycles of 94 °C for 30 s, 60 °C for 30 s, and 72 °C for 30 s. Fluorescence was recorded during each annealing step. At the end of each PCR run, the data were automatically analyzed by the system, and the amplification plots were obtained. The expression levels of the genes were normalized to an endogenous *gapdh* cDNA.

### Immunofluorescence

The freshly dissected brain from pups at P1, P7, and P21 was fixed in 4% paraformaldehyde in 0.1 M phosphate buffer (pH 7.4) for 24 h at 4 °C. They were subsequently dehydrated in 10, 20, and 30% sucrose dissolved in 0.1 M phosphate buffer (pH 7.4). The brain was sectioned into 12-μm slices in a cryostat. After washing in PBS, the slices were blocked in PBS containing 0.1% triton X-100, 5% newborn goat serum, and 5% horse serum for 1 h. The primary antibodies were diluted in PBS before added to the slices. After incubation at 4 °C for 16 h, the sections were washed with PBS, and the secondary antibodies were added and incubated at 4 °C for 16 h. The slices were then washed in PBS and counterstained with 0.1 g/ml Hoechst 33342 solution. Antibodies used in immunofluorescence were anti-NeuN antibody (1:1000; proteintech); anti-CaMKIV antibody (1:1000; proteintech); anti-CREB antibody (1:1000; proteintech); anti-MAP2 (1:500; abcam); donkey-anti-mouse Alexa 488; and and goat-anti-rabbit Cy3.

### Statistical analysis

Statistical analysis used GraphPad Prism 8 software (San Diego, CA, USA). Normality and homoscedasticity of the data were performed using Levene’s test. Independent sample *t* test and one-way analysis of variance followed by Bonferroni’s post hoc comparisons tests were utilized for comparisons for different groups. All data were presented as mean ± standard deviation. Two-sided *P* value <0.05 was considered statistically significant.

## Supplementary information

tableS1

## Data Availability

The datasets used and/or analyzed during the current study are available from the corresponding author on reasonable request.
